# Supplementary material for: Sleep Disorders in a Sample of Patients with Pediatric-Onset Multiple Sclerosis: Focus on Restless Legs Syndrome
Source: J Clin Med. 2025 May 2;14(9):3157. doi: 10.3390/jcm14093157 (PMC12072441; doi:10.3390/jcm14093157)
Supplement: Supplementary file 1 [file jcm-14-03157-s001.zip › jcm-3533043-supplementary.pdf]

Supplementary Information

Sleep disorders in a sample of patients with Pediatric Onset Multiple Sclerosis: focus on Restless Leg Syndrome

Elena Panella [1]*, Laura Papetti[2], Martina Proietti Checchi[2], Samuela Tarantino[2], Michela Ada Noris Ferilli[2], Gabriele Monte[2], Alessandra Voci[2], Claudia Ruscitto[2], Luigi Mazzone[1], Massimiliano Valeriani[1,2,3], Romina Moavero[1,2]

1 Child Neurology and Psychiatry Unit, Systems Medicine Department, Tor Vergata University of Rome, 00133 Rome, Italy;
2 Developmental Neurology Unit, Bambino Gesù Children's Hospital, IRCCS, 00165 Rome, Italy;
3 Translational Pain Neuroscience and Precision Medicine, CNAP, Dept. of Health Science and Technology, School of Medicine, Aalborg University.
* Correspondence: elena.panella3@gmail.com

Academic Editor: Giuseppe Signoriello

Received: 1 March 2025
Revised: 29 April 2025
Accepted: 30 April 2025
Published: 2 May 2025

Citation: Panella, E.; Papetti, L.; Proietti Checchi, M.; Tarantino, S.; Ferilli, M.A.N.; Monte, G.; Voci, A.; Ruscitto, C.; Mazzone, L.; Valeriani, M.; et al. Sleep Disorders in a Sample of Patients with Pediatric-Onset Multiple Sclerosis: Focus on Restless Legs Syndrome. *J. Clin. Med.* **2025**, *14*, 3157. https://doi.org/10.3390/jcm14093157

Copyright: © 2025 by the authors. Submitted for possible open access publication under the terms and conditions of the Creative Commons Attribution (CC BY) license (https://creativecommons.org/licenses/by/4.0/).

Table S1: IRLSSG consensus diagnostic criteria for RLS

Diagnosis of RLS requires of all the following features:

1. An urge to move the legs usually but not always accompanied by or felt to be caused by uncomfortable and unpleasant sensations in the legs (a,b)
2. The urge to move the legs and any accompanying unpleasant sensations begin or worsen during periods of rest or inactivity such as lying down or sitting
3. The urge to move the legs and any accompanying unpleasant sensations are partially or totally relieved by movement, such as walking or stretching, at least as long as the activity continues (c)
4. The urge to move the legs and any accompanying unpleasant sensations during rest or inactivity only occur or are worse in the evening or night than during the day (d)
5. The occurrence of the above features are not solely accounted for as symptoms primary to another medical or a behavioral condition (e.g., myalgia, leg cramps, positional discomfort, habitual foot tapping) (e)

Specifier for clinical significance of RLS
The symptoms of RLS cause significant distress or impairment in social, occupational, educational, or other important areas of functioning by the impact on sleep, energy/vitality, daily activities, behavior, cognition, or mood

Specifiers for clinical course of RLS (f)
A. Chronic-persistent RLS: symptoms when not treated would occur on average at least twice weekly for the past year
B. Intermittent RLS: symptoms when not treated would occur on average <2/week for the past year, with at least five lifetime events

(a) Sometimes the urge to move the legs is present without the uncomfortable sensations and sometimes the arms or other parts of the body are involved in addition to the legs.
(b) For children, the description of these symptoms should be in the child's own words
(c) When symptoms are very severe, relief by activity may not be noticeable but must have been previously present.
(d) When symptoms are very severe, the worsening in the evening or night may not be noticeable but must have been previously present.
(e) These conditions, often referred to as "RLS mimics," have been commonly confused with RLS, particularly in surveys because they produce symptoms that meet or at least come close to meeting criteria 1–4 above. The list here gives some examples that have been noted as particularly significant in epidemiologic studies and clinical practice. However, RLS may also occur with any of these conditions, requiring a clear delineation of the RLS feelings from the other sensations.
(f) f The clinical course criteria do not apply for pediatric cases or for some special cases of provoked RLS such as pregnancy or drug-induced RLS, in which the frequency may be high but limited to the duration of the provocative condition.

Abbreviations: IRLSSG International Restless Leg Syndrome Study Group; RLS: Restless Leg Syndrome

Table S2: Demographic characteristic and scores of Sleep questionnaires

	SDSC	PSQI
Patients, n°	26	15
Age y, mean±SD (range)	15±2,8 (7,3-17,8)	19,8±1,1 (18-22,5)
Presence of SD, n° (%)	17 (65,3%)	8 (53,3%)
Tot score, mean±SD (range)	46,3±12,4 (28-75)	6,4±3,1 (2-14)

Abbreviations: SDSC: Sleep Disturbance Scale for Children; PSQI: Pittsburgh Sleep Quality Index; SD: Standard deviation

Table S3: Comparison of demographic and clinical features between Good and Bad Sleepers

	Good sleepers (n.16, 39%)			Bad sleepers (n.25, 61%)			P value
	Mean	SD	Range	Mean	SD	Range	
Age, y	16.5	3.5	10-22	17	3.2	7-22	0.61
Age at disease onset, y	13.2	3.3	7-16	14	2.6	5-17	0.35
Disease duration, m	45.1	31.2	8-113	45.7	32.9	6-130	0.96
Relapse rate	0.19	0,4	0-1	0.13	0.3	0-1	0.59
EDSS	0.06	0,2	0-1	0.4	0.8	0-3	0.074
Days at hospital	10.8	4.3	6-21	11.8	5.8	0-22	0.58
Total exposure time to DMT,m	42.1	24.6	17-100	41.1	26	7-108	0.65

Abbreviations: SD: Standard Deviation; EDSS: Expanded Disability Status Scale
